# Minimally Invasive Plate Osteosynthesis for a Distal Radius Fracture with Forearm Skin Problem

**DOI:** 10.1155/2018/8195376

**Published:** 2018-06-24

**Authors:** Kiyohito Naito, Yoichi Sugiyama, Mayuko Kinoshita, Ahmed Zemirline, Chihab Taleb, Thitinut Dilokhuttakarn, Philippe Liverneaux, Kazuo Kaneko

**Affiliations:** ^1^Department of Orthopaedics, Juntendo University School of Medicine, Tokyo, Japan; ^2^Centre de la Main de Bretagne, Centre Hospitalier de Saint Grégoire, Saint-Grégoire, France; ^3^Unité de Chirurgie de la Main et du Poignet, Groupe Hospitalier de Mulhouse, Mulhouse, France; ^4^Department of Orthopedics, Srinakharinwirot University, Nakhon Nayok, Thailand; ^5^Department of Hand Surgery, SOS Main, CCOM, University Hospital of Strasbourg, FMTS, University of Strasbourg, Icube CNRS 7357, 10 Avenue Baumann, 67400 Illkirch, France

## Abstract

In this study, we performed osteosynthesis for a distal radius fracture using a minimally invasive approach for a patient with skin disorder of the forearm and obtained favorable results. This case report may provide new findings confirming the usefulness of this surgical approach for distal radius fractures. Blister formation on the right forearm was observed in a 53-year-old female who was diagnosed with a distal fracture of the right radius and underwent splinting in a local hospital, and she was referred to our hospital 2 days after the injury. Minimally invasive locking plate osteosynthesis was performed, and there was no skin lesion at this incision site. Postoperatively, there were no complications in soft tissues and the operative scar was almost unrecognizable. We reported volar locking plate osteosynthesis using the minimally invasive approach in a patient with skin disorder of the forearm. Such patients are rarely encountered. However, this minimally invasive approach is extremely useful for utilizing the advantages of volar locking plate fixation without being affected by the soft tissue environment.

## 1. Introduction

In recent years, there have been some studies on a minimally invasive approach in volar locking plate osteosynthesis for distal radius fractures [1, 2]. The feasibility of plate fixation using the Henry approach (10 mm) was already reported [3].

In this study, we performed osteosynthesis for a distal radius fracture using a minimally invasive approach for a patient with skin disorder of the forearm and obtained favorable results. This case report may provide new findings confirming the usefulness of this surgical approach for distal radius fractures.

## 2. Case Presentation

**Figure 1:**
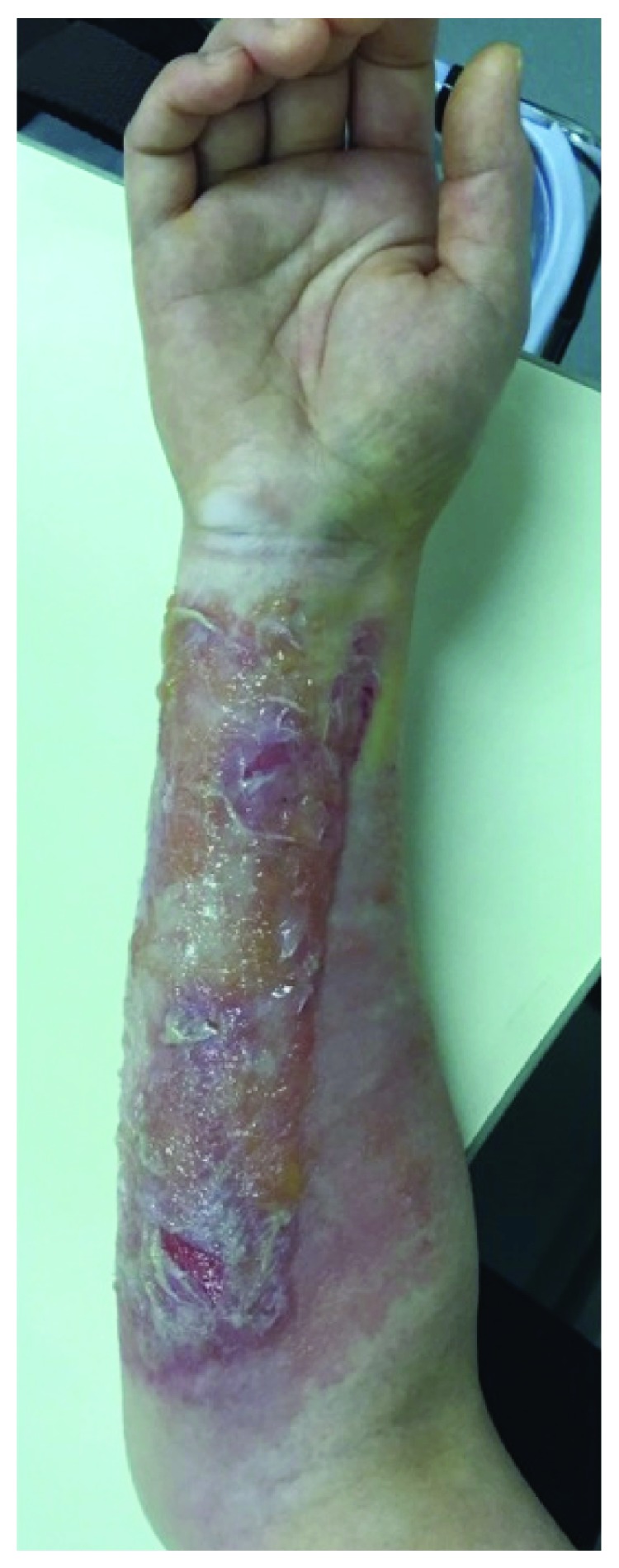
The forearm skin state on her visit to our hospital. Skin disorder accompanied by blister formation was observed on the right forearm. The blistering was present along the splint application area.

**Figure 2:**
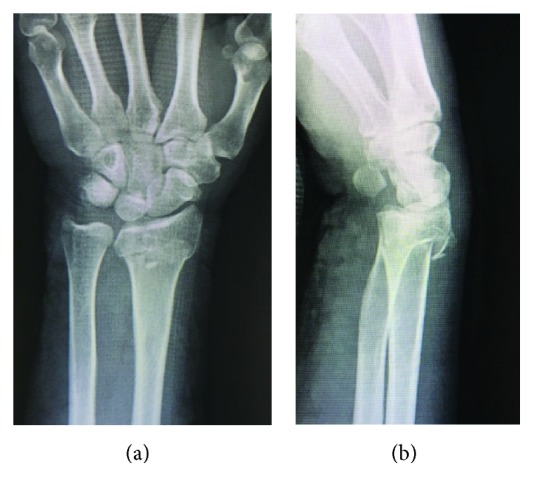
Plain X-ray films on her visit to our hospital. (a) Frontal plain X-ray image. (b) Lateral plain X-ray image. Plain X-ray images showed a distal radius fracture accompanied by dorsal displacement of the distal bone fragment. Due to the skin state, conservative treatment by external fixation, such as splinting and casting, was difficult. Surgery after improvement of the skin state was considered to be more invasive due to bone union. Therefore, osteosynthesis using a minimally invasive approach was planned.

A 53-year-old female with no previous history visited a local hospital due to right wrist pain and swelling caused by falling. She was diagnosed with a distal fracture of the right radius, underwent splinting, and returned home. When she visited the local hospital again 2 days after the injury, blister formation on the right forearm was observed, and she was referred to our hospital. The blister was observed along the splint application area (Figure 1) and was considered to be due to the heat and stuffiness of the splint. Plain X-ray examination revealed a distal radius fracture accompanied by dorsal displacement of the distal bone fragment (AO classification: type A2) (Figures 2(a) and 2(b)). Based on the skin condition, we considered conservative treatment by external fixation using a splint or cast to be difficult, and surgery after improvement of the skin state would be more invasive due to bone union and, therefore, planned minimally invasive locking plate osteosynthesis.

**Figure 3:**
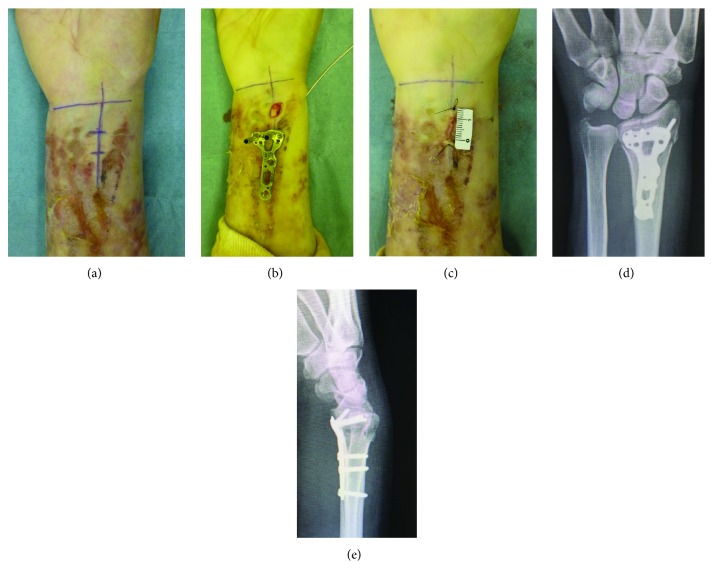
Findings during operation. (a) Skin incision design. This surgical technique could be performed because there was no skin lesion at the skin incision site. (b, c) Volar locking plate fixation. After reduction of the distal bone fragment using a Kirschner wire, osteosynthesis was performed using a volar locking plate (Acu-Loc 2 proximal plate standard, Nihon Medical Next, Osaka, Japan). (d, e) Plain X-ray images after volar locking plate fixation ((d): frontal image, (e): lateral image). Fixation in a favorably reduced position is observed.

As we previously reported, surgery was performed using the Henry approach through a 10 mm incision starting from 15 mm proximal to the radial styloid process at 9 days after injury [3]. In this patient, there was no skin lesion at this incision site, which allowed this surgical technique (Figure 3(a)). After reduction of the distal bone fragment using a Kirschner wire, osteosynthesis was performed using a volar locking plate (Acu-Loc 2 proximal plate standard, Nihon Medical Next, Osaka Japan) (Figures 3(b) and 3(c)). After the operation, a favorable alignment was obtained (Figures 3(d) and 3(e)). The wrist was immobilized postoperatively in a bulky dressing without an arm splint until the tissue swelling had decreased.

All muscles, vessels, and nerves of the anterior compartment—except the radial artery—were retracted ulnarly. The pronator quadratus was incised transversely at its distal portion and dissected off the periosteum using a periosteal elevator preserving its ulnar and radial insertions. Therefore, active finger motion was encouraged immediately after the operation and wrist mobilization was started as soon, and as much, as pain allowed. Moreover, this approach can avoid the median nerve damages during the surgery.

**Figure 4:**
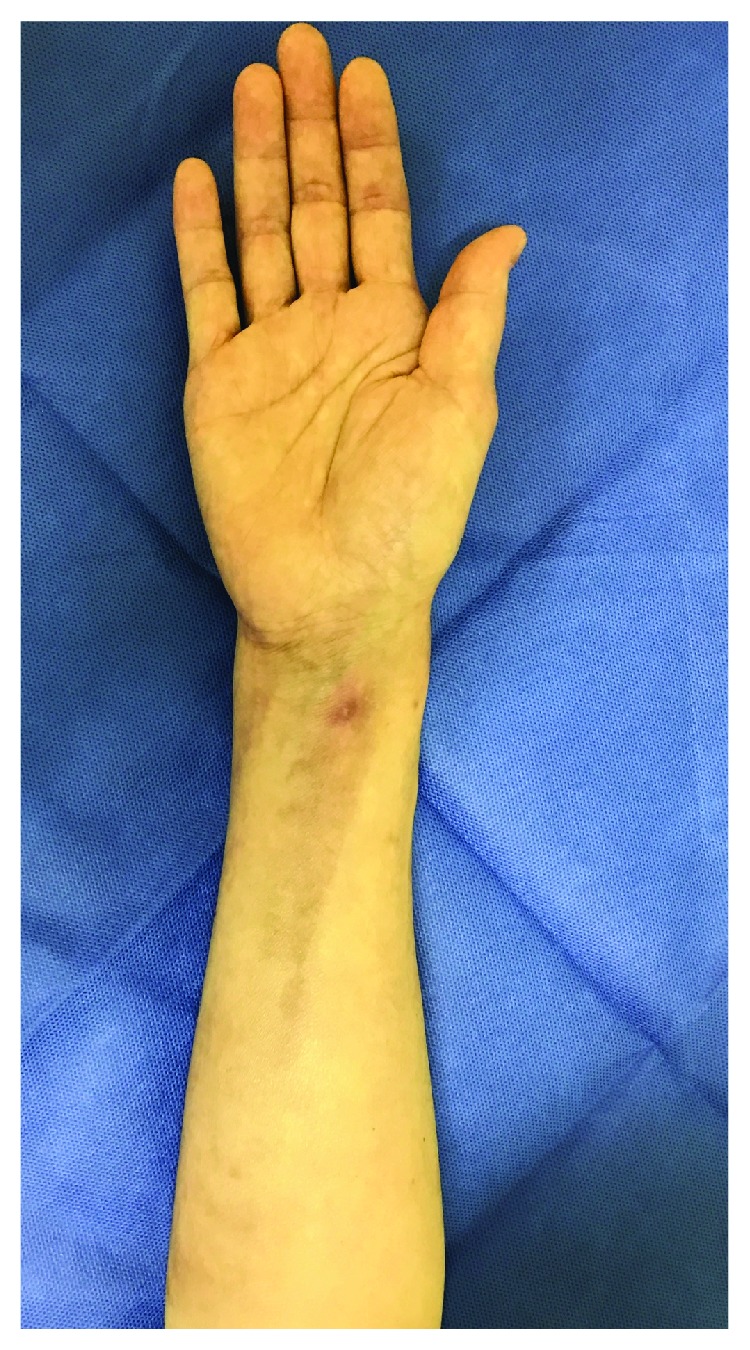
Forearm skin state 6 months after the operation. The forearm skin state and surgical wound favorably improved, and she returned to her preinjury job without any problems in daily life.

Six months after the operation, favorable union of the radius was obtained. The wrist range of motion was as follows: flexion, 70°; extension, 65°; pronation, 85°; and supination, 85°. The visual analogue scale (VAS) was 1/10, and the quick disabilities of the arm, shoulder, and hand (Q-DASH) score was 20.45/100. The Mayo wrist score was 85/100 (excellent). The state of the forearm skin and surgical wound favorably improved (Figure 4), and she returned to her preinjury job.

## 3. Discussion

Previous studies on volar locking plate osteosynthesis using the minimally invasive approach for distal radius fractures have demonstrated some advantages (the patients' satisfaction level and aesthetically pleasing wound healing) of this technique, but clinical results were similar to those after conventional surgical methods [3, 4]. Authors have reported that there is a wide acceptable range of the reduced position, and anatomical reduction is not always required [5–7]. We have actively used the minimally invasive approach and believe that the advantages of this approach should be reevaluated.

In general, distal radius fractures are conservatively treated by manual reduction and casting [8]. However, this conservative treatment is not indicated for limbs with skin lesions. We previously treated distal radius fractures in limbs containing shunts in dialysis patients with skin fragility using volar locking plate as well as this case [9]. In patients with skin fragility, conservative treatment by splinting or casting is difficult [10, 11]. In addition, percutaneous pinning or external fixation may increase aggravation of skin lesions and infection [12]. Of course, it was also possible to place an external fixator. But, discharge from pin site may continue and this can lead to infection. When priority is given to the avoidance of soft tissue damage, volar locking plate fixation is the only method with fixation force allowing early joint motion at present. From our experience with distal radius fractures patients in limbs containing shunts, we learned that volar locking plate fixation for distal radius fractures is useful and possible by protective manipulation of soft tissue including the skin during operation [9]. This experience was applied to the present case. As shown in Figures 3(a) and 3(b), this surgical technique was possible because there was no skin lesion 15–25 mm proximal to the radial styloid process, which was the skin incision site for the insertion of a volar locking plate.

We speculated about the development of the skin disorder in this patient. Some patients with skin disorders due to splinting and casting have been reported [10, 13]. Blister formation and burns at moderate temperatures are widely known and have recently been applied to the production of animal models of burn injury [14]. We speculate that the moisture from the used splint could not be adequately removed, and heat generated by the splint caused blister formation in this patient. Although close examination was performed for suspected contact dermatitis and allergic reactions, no abnormality was detected. Close examination for metal allergy was also performed before surgery to confirm that the implant used in this study could be safely used. During the clinical course of this patient, no aggravation of the skin disorder was observed, and the skin state steadily improved after removal of the physical stimulus.

We reported volar locking plate osteosynthesis using the minimally invasive approach in a patient with skin disorder of the forearm. Such patients are rarely encountered. However, this minimally invasive approach is extremely useful for utilizing the advantages of volar locking plate fixation without being affected by the soft tissue environment. This operative procedure is used in relatively stable fracture type. Fracture types which the authors believe this indication are A2 and A3 (extra-articular fractures) and C1 and C2 (complete articular fractures) according to AO classification. Even if type C1 or C2 fractures, the fractures that involve the depressed intra-articular fragment which is needed to the intramedullary reduction to perform osteosynthesis by this technique are not suitable. Moreover, type B fractures (partial articular fractures) are also unsuitable in our consideration [3].
